# Elevated plasma sclerostin is associated with high brain amyloid-β load in cognitively normal older adults

**DOI:** 10.1038/s41514-023-00114-4

**Published:** 2023-09-04

**Authors:** Jun Yuan, Steve Pedrini, Rohith Thota, James Doecke, Pratishtha Chatterjee, Hamid R. Sohrabi, Charlotte E. Teunissen, Inge M. W. Verberk, Erik Stoops, Hugo Vanderstichele, Bruno P. Meloni, Christopher Mitchell, Stephanie Rainey-Smith, Kathryn Goozee, Andrew Chi Pang Tai, Nicholas Ashton, Henrik Zetterberg, Kaj Blennow, Junjie Gao, Delin Liu, Frank Mastaglia, Charles Inderjeeth, Minghao Zheng, Ralph N. Martins

**Affiliations:** 1 Perron Institute for Neurological and Translational Science, Nedlands, WA Australia; 2 Centre for Orthopaedic Translational Research, Medical School, The University of Western Australia, Nedlands, WA Australia; 3 School of Medical and Health Sciences, Edith Cowan University, Joondalup, WA Australia; 4 Department of Biomedical Sciences, Macquarie University, North Ryde, NSW Australia; 5 Australian E-Health Research Centre, CSIRO, Brisbane, QLD Australia; 6 Centre for Healthy Ageing, Health Future Institute, Murdoch University, Perth, WA Australia; 7 The Centre of Excellence for Alzheimer’s Disease Research and Care, Edith Cowan University, Joondalup, WA Australia; 8 Neurochemistry Laboratory, Department of Clinical Chemistry, Amsterdam Neuroscience, Neurodegeneration, Amsterdam UMC, Vrije Universiteit Amsterdam, Amsterdam, The Netherlands; 9 ADx NeuroSciences, Technologiepark 94, 9052 Gent, Belgium; 10 Biomarkable BV, Gent, Belgium; 11 KaRa Institute of Neurological Disease, Macquarie Park, NSW Australia; 12 Department of Psychiatry and Neurochemistry, Institute of Neuroscience and Physiology, the Sahlgrenska Academy at the University of Gothenburg, Mölndal, Sweden; 13 Wallenberg Centre for Molecular and Translational Medicine, University of Gothenburg, Mölndal, Sweden; 14 Clinical Neurochemistry Laboratory, Sahlgrenska University Hospital, Mölndal, Sweden; 15 Department of Neurodegenerative Disease, UCL Institute of Neurology, Queen Square, London, UK; 16 UK Dementia Research Institute at UCL, London, UK; 17 Hong Kong Center for Neurodegenerative Diseases, Clear Water Bay, Hong Kong, China; 18 Department of Orthopaedic Surgery, Shanghai Jiao Tong University Affiliated Sixth People’s Hospital, Shanghai, China; 19 School of Medicine, The University of Western Australia, Perth, WA Australia; 20 Sir Charles Gairdner and Osborne Park Health Care Group, Perth, Australia

**Keywords:** Biomarkers, Neuroscience

## Abstract

Osteoporosis and Alzheimer’s disease (AD) mainly affect older individuals, and the possibility of an underlying link contributing to their shared epidemiological features has rarely been investigated. In the current study, we investigated the association between levels of plasma sclerostin (SOST), a protein primarily produced by bone, and brain amyloid-beta (Aβ) load, a pathological hallmark of AD. The study enrolled participants meeting a set of screening inclusion and exclusion criteria and were stratified into Aβ− (*n* = 65) and Aβ+ (*n* = 35) according to their brain Aβ load assessed using Aβ-PET (positron emission tomography) imaging. Plasma SOST levels, apolipoprotein E gene (*APOE*) genotype and several putative AD blood-biomarkers including Aβ40, Aβ42, Aβ42/Aβ40, neurofilament light (NFL), glial fibrillary acidic protein (GFAP), total tau (t-tau) and phosphorylated tau (p-tau181 and p-tau231) were detected and compared. It was found that plasma SOST levels were significantly higher in the Aβ+ group (71.49 ± 25.00 pmol/L) compared with the Aβ− group (56.51 ± 22.14 pmol/L) (*P* < 0.01). Moreover, Spearman’s correlation analysis showed that plasma SOST concentrations were positively correlated with brain Aβ load (ρ = 0.321, *P* = 0.001). Importantly, plasma SOST combined with Aβ42/Aβ40 ratio significantly increased the area under the curve (AUC) when compared with using Aβ42/Aβ40 ratio alone (AUC = 0.768 vs 0.669, *P* = 0.027). In conclusion, plasma SOST levels are elevated in cognitively unimpaired older adults at high risk of AD and SOST could complement existing plasma biomarkers to assist in the detection of preclinical AD.

## Introduction

Increasing age is a major risk factor for both osteoporosis and Alzheimer’s disease (AD), and clinical outcomes reveal underlying associations between the two disorders including the observation that patients with osteoporosis are prone to develop AD in their later life^1,2^. Emerging evidence illustrates that bone can influence the function of other organs by secreting various proteins that enter the circulation^3^. In particular, osteocalcin which is exclusively produced by osteoblasts has been demonstrated to regulate brain development and play a role in age-related cognition decline^4–6^, and lower osteocalcin is indicated to be an independent predictor of worse cognitive performance^7^. Nevertheless, whether bone-derived factors are associated with AD pathological alterations is unknown.

We previously reviewed several bone-derived proteins with the potential to be involved in the pathogenesis of AD, and sclerostin (SOST) which is mainly produced by osteocytes, was one of the candidate proteins discussed^8^. Sclerostin is encoded by the *Sost* gene and is one of the major antagonists of Wnt signaling^9^. Interestingly, circulating SOST protein levels increase with increasing age^10^ and it has been proposed as a candidate senescence marker^11^. Moreover, dysfunction of Wnt/β-catenin signaling has been implicated in AD pathological mechanisms of amyloid beta (Aβ) plaque formation. Specifically, inhibition of the Wnt signaling can promote the amyloidogenic processing of Aβ precursor protein (APP) and thereby leads to an enhanced production of the Aβ42 peptide, resulting in a higher Aβ42/Aβ40 ratio and Aβ oligomer levels^12^. In contrast, activation of Wnt signaling decreased Aβ42 levels while increasing Aβ40 levels, led to a reduced Aβ42/Aβ40 ratio. However, a direct involvement of SOST, as a Wnt signaling inhibitor, in AD pathogenesis has yet to be established^12^.

Specific proteins in blood have been employed as biomarkers to predict the preclinical stage of AD (cognitively unimpaired while proteinopathies develop), such as: Aβ42, Aβ40, Aβ42/Aβ40, glial fibrillary acidic protein (GFAP), total tau (t-tau), phosphorylated tau (p-tau181, p-tau231), and neurofilament light (NFL)^13–20^. Given the fact that SOST is a Wnt signaling inhibitor and its levels in the blood are elevated with age, we hypothesize that plasma SOST is associated with early AD pathological change, i.e., cerebral Aβ deposition, and could be a potential predictive blood biomarker for AD in individuals “at risk”. In the current study, we employed a cohort of cognitively healthy participants divided into Aβ− and Aβ+ groups according to their brain Aβ load based on PET imaging assessment. To examine the association between plasma SOST and the presence of brain amyloidosis, plasma SOST concentrations were compared between Aβ− and Aβ+ participants before and after stratification by sex and apolipoprotein E (*APOE*) gene ε4 carriage. In addition, we evaluated the correlation between plasma SOST levels and brain Aβ load reflected by the mean standard uptake value ratio (SUVR) in different cortical regions. Furthermore, we conducted assessment of the correlation between plasma SOST and AD biomarkers including Aβ40, Aβ42, Aβ42/Aβ40 ratio, NFL, GFAP, t-tau, p-tau181 and p-tau231, as well as parallel comparisons of the diagnostic accuracy of plasma SOST with these blood biomarkers. Lastly, we evaluated the potential of plasma SOST as a predictor in distinguishing Aβ+ from Aβ− individuals.

## Results

### Cohort characteristics

Brain imaging and blood samples were obtained from 100 participants, with demographic and clinical characteristics summarized in Table 1. No significant difference was observed in terms of age, sex, MMSE and BMI between the Aβ− and Aβ+ groups. As expected, there was a higher ratio of *APOE* ε4 carriage in the Aβ+ group (45.7%) compared with Aβ− group (7.7%; *P* < 0.001). By design, the SUVR values in the Aβ+ group (1.71 ± 0.26) were significantly higher than the Aβ− group (1.16 ± 0.09; *P* < 0.001). Of the biomarkers assessed in the present study, significant differences of the values of GFAP, p-tau181, p-tau231 and Aβ42/Aβ40 ratio were found between the groups (Table 1).

**Table 1:** Cohort demographic characteristics.

	Aβ−	Aβ+	*P*
Age (mean ± SD)	77.62 ± 5.56	79.23 ± 5.38	0.165
Sex (M/F)	19/46	13/22	0.419
*APOE* ε4 carriers (n (%))	5 (7.7)	16 (45.7)	**<0.001**
SUVR (mean ± SD)	1.16 ± 0.09	1.71 ± 0.26	**<0.001**
MMSE (mean ± SD)	28.51 ± 1.16	28.80 ± 1.11	0.219
BMI (mean ± SD)	27.39 ± 4.48	28.05 ± 4.74	0.494
GFAP (pg/mL, mean ± SD)	145.5 ± 49.2	210.5 ± 83.9	**<0.001**
NFL (pg/mL, mean ± SD)	19.36 ± 9.00	21.02 ± 10.09	0.416
t-tau (pg/mL, mean ± SD)	1.19 ± 0.37	1.34 ± 0.39	0.068
p-tau181 (pg/mL, mean ± SD)	13.55 ± 5.69	17.27 ± 5.58	**0.003**
p-tau231 (pg/mL, mean ± SD)	11.39 ± 6.45	19.08 ± 7.16	**<0.001**
Aβ42 (pg/mL, mean ± SD)	21.96 ± 4.58	19.54 ± 5.74	**0.041**
Aβ40 (pg/mL, mean ± SD)	95.39 ± 14.75	98.37 ± 16.88	0.395
Aβ42/40 (mean ± SD)	0.23 ± 0.04	0.20 ± 0.05	**0.003**

Baseline characteristics including sex, age, body mass index (BMI), *APOE* ε4 status, Mini-mental State Examination (MMSE) scores, brain amyloid-beta (Aβ) load represented by the standard uptake value ratio (SUVR) of ligand 18F-Florbetaben (FBB) in the neocortical region normalized with that in cerebellum, and blood-based biomarkers (GFAP, NFL, t-tau, p-tau181, p-tau231, Aβ42, Aβ40 and Aβ42/40) have been compared between Aβ− (SUVR < 1.35, *n* = 65) and Aβ+ (SUVR ≥ 1.35, *n* = 35) participants. Student’s *t* test or Chi-square tests were employed as appropriate. Data are presented in mean ± SD, and *P* values in bold font were considered as significant (*P* < 0.05). (*N* = 100, Aβ− *n* = 65, Aβ+ *n* = 35).

*GFAP* glial fibrillary filament protein, *NFL* neurofilament light.

### Associations of AD-related risk factors including age, sex and *APOE* allele status with plasma SOST

**Fig. 1:**
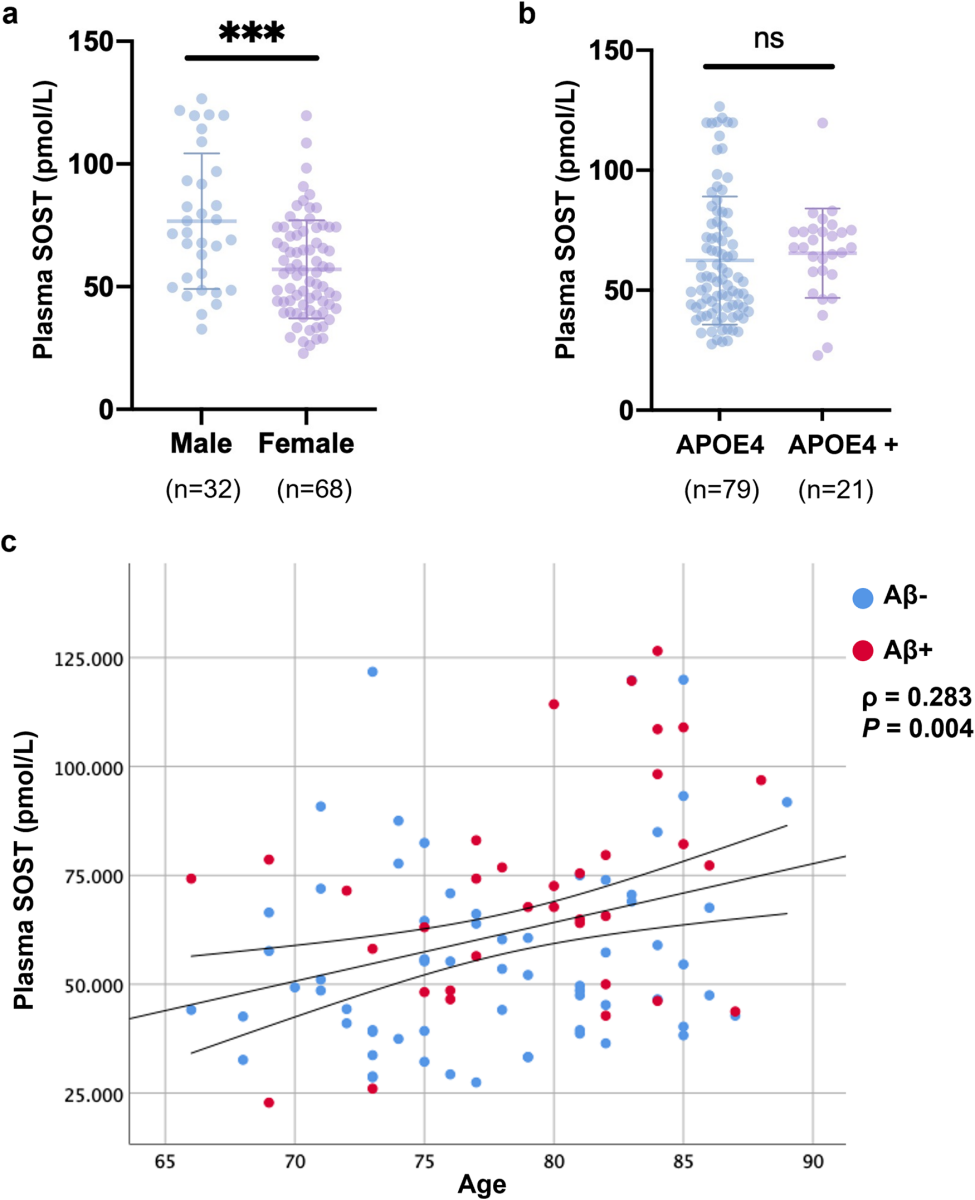
Associations of AD-related risk factors, age, sex and *APOE*4 allele status with plasma sclerostin. **a** Plasma sclerostin levels were compared between male and female participants. **b** Plasma sclerostin levels were compared between *APOE*4– and *APOE4*+ participants. **c** Correlations between plasma sclerostin levels and age. The line segment within each jitter plot represents the median of the data and error bars in the graphs represent the interquartile range for the groups compared. ***P ≤ 0.001, ns no significant difference. Correlation coefficients and *P* values were calculated using Spearman’s correlation coefficient (ρ).

Plasma SOST levels were higher in males (76.66 ± 27.67 pmol/L), compared with females (54.73 ± 18.74 pmol/L; *P* < 0.001; Fig. 1a). Plasma SOST levels did not differ significantly in *APOE* ε4 non-carriers and *APOE* ε4 carriers (non-carriers = 62.45 ± 25.76 pmol/L; carriers = 59.12 ± 16.98 pmol/L; *P* = 0.57; Fig. 1b). Consistent with previous studies^10^, plasma SOST levels positively correlated with age (ρ = 0.283, *P* = 0.004; Fig. 1c).

### Comparison of plasma SOST between Aβ− participants and Aβ+ participants

Plasma SOST levels were significantly higher in the Aβ+ group (71.49 ± 25 pmol/L) compared with the Aβ− group (56.51 ± 22.14 pmol/L), before and after adjusting for potential AD risk factors including age, sex and *APOE* ε4 status (*P* < 0.003, P* = 0.008; Table 2). On stratifying study participants by *APOE* ε4 carriage status (ε4 non-carriers: *n* = 79, and ε4 carriers: *n* = 21), significantly higher plasma SOST levels were observed in the *APOE* ε4 non-carrier Aβ+ group (*n* = 19, 80.93 ± 27.18 pmol/L) compared with the *APOE* ε4 non-carrier Aβ− group (*n* = 60, 55.94 ± 22.63 pmol/L), before and after adjusting for potential risk factors including age and sex (*P* < 0.001, P* = 0.001; Table 2). Within the *APOE* ε4 carriers, no significant difference in plasma SOST levels was observed between the Aβ+ group (*n* = 16, 60.42 ± 16.98 pmol/L) and the Aβ− group (*n* = 5, 63.42 ± 15.06 pmol/L, *P* = 0.728, P* = 0.368; Table 2). This observation could be attributed to the small sample size of the *APOE* ε4 subset available within the present study.

**Table 2:** Comparison of plasma sclerostin within all Aβ− and Aβ+ participants and subgroups stratified by *APOE* ε4 status and sex, respectively.

	Aβ−	Aβ+	*P*	P*
SOST in all participants	56.51 ± 22.14	71.49 ± 25.00	**0.003**	**0.008**
SOST in *APOE* ε4 non-carriers	55.94 ± 22.63	80.93 ± 27.18	**<0.001**	**0.001**
SOST in *APOE* ε4 carriers	63.42 ± 15.06	60.42 ± 16.98	0.728	0.368
SOST in males	69.55 ± 28.18	87.07 ± 24.28	0.078	0.050
SOST in females	51.12 ± 16.68	62.28 ± 20.87	**0.020**	**0.033**

Student’s *t* test or Chi-square tests were employed as appropriate. Data are presented in mean ± SD in pmol/L, and *P* values in bold font were considered as significant (*P* < 0.05). P* represents *P* values adjusted for age, sex and *APOE* ε4 status.

When stratifying study participants by sex (male: *n* = 32; female: *n* = 68), there was a trend of higher plasma SOST levels in male Aβ+ participants (*n* = 13, 87.07 ± 24.28 pmol/L) compared with male Aβ− participants (*n* = 19, 69.55 ± 28.18 pmol/L, *P* = 0.078, P* = 0.05; Table 2). It is likely that an increased sample size of male participants would uncover a statistically significant effect. In females, a significantly higher plasma level of SOST was observed in Aβ+ participants (*n* = 22, 62.28 ± 20.87 pmol/L) compared with Aβ− participants (*n* = 46, 51.12 ± 16.68 pmol/L), before and after adjusting for potential risk factors including age and *APOE* ε4 status (*P* = 0.02, P* = 0.033; Table 2).

### Correlation of plasma SOST with selected AD biomarkers

We next assessed whether plasma SOST levels correlate with any AD-related biomarkers using Spearman’s correlation. We found a significant association between SOST levels and NFL, GFAP, phosphorylated versions of the tau protein (p-tau181 and p-tau231) and Aβ40, while no significant association was found between SOST levels and other AD-related biomarkers (Table 3, unadjusted correlations are shown in Supplementary Fig. 1).

**Table 3:** Correlations between plasma sclerostin levels and AD biomarkers among all participants and subgroups stratified by brain Aβ status.

	All participants		Aβ−		Aβ+	
	ρ	*P*	ρ	*P*	ρ	*P*
NFL	0.337	**<0.001**	0.445	**<0.001**	0.095	0.587
GFAP	0.314	**0.001**	0.218	0.081	0.067	0.703
t-tau	−0.017	0.865	−0.018	0.889	−0.267	0.121
p-tau181	0.393	**<0.001**	0.279	**0.025**	0.143	0.434
p-tau231	0.316	**0.002**	0.241	0.053	0.114	0.541
Aβ42	0.097	0.348	0.217	0.090	0.061	0.737
Aβ40	0.283	**0.005**	0.336	**0.007**	0.153	0.395
Aβ42/40	−0.155	0.133	−0.076	0.559	−0.128	0.479

Unadjusted correlation coefficients (ρ) and *P* values between plasma sclerostin and AD-related biomarkers were calculated using Spearman’s correlation analysis.

*P* values in bold font were considered as significant (*P* < 0.05).

The cohort was then further stratified by brain Aβ deposition status. It was found that SOST levels were significantly correlated with NFL, p-tau181 and Aβ40 while no correlation was observed with other biomarkers in Aβ− group. For individuals with Aβ+ status, no significant association was found between SOST levels and all the selected biomarkers (Table 3).

### Evaluation of plasma SOST as a predictor for brain Aβ status

**Fig. 2:**
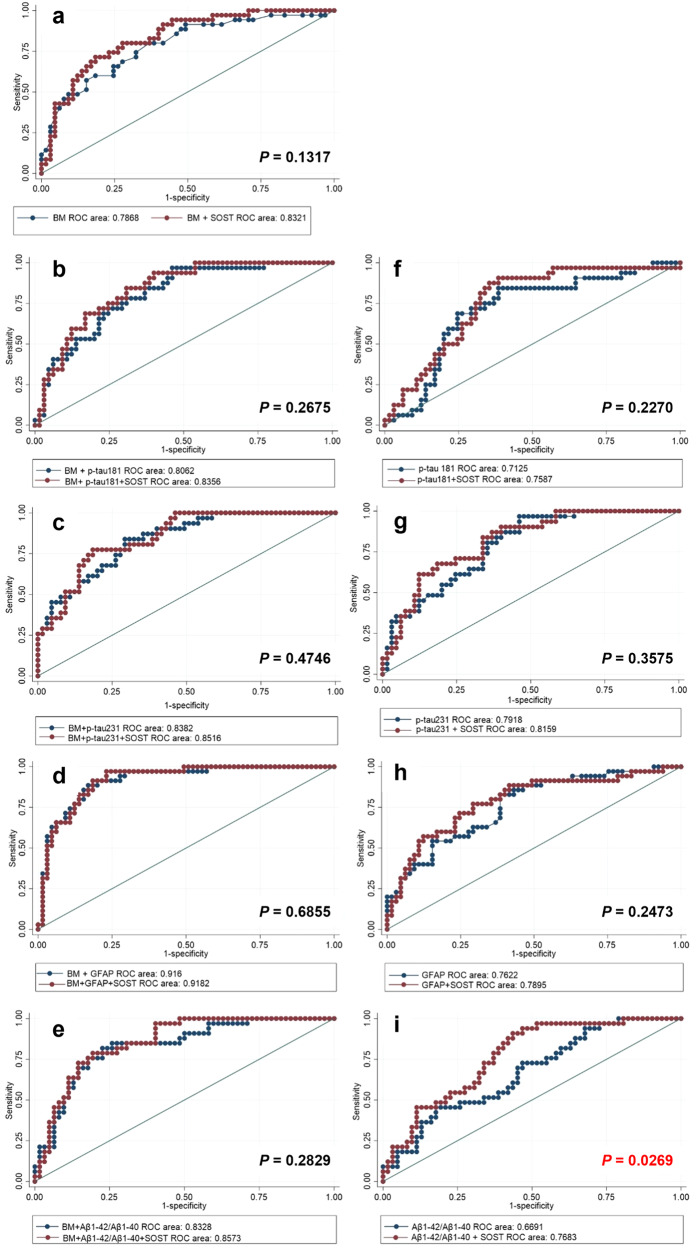
Receiver operating characteristic curves for distinguishing Aβ+ individuals from Aβ− individuals using plasma sclerostin in combination with the base model (BM) and/or AD-related biomarkers. A base model (BM) including age, sex and *APOE* ε4 carriage status was included in the analysis. Receiver operating characteristic (ROC) curves are presented with the BM (**a**–**e**) or without (**f**–**i**). **a** (BM vs BM + SOST), **b** (BM + p-tau181 vs BM + p-tau181 + SOST), **c** (BM + p-tau231 vs BM + p-tau231 + SOST), **d** (BM + GFAP vs BM + GFAP + SOST), **e** (BM + Aβ1-42/Aβ1-40 vs BM + Aβ1-42/Aβ1-40 + SOST), **f** (p-tau181 vs p-tau181 + SOST), **g** (p-tau231 vs p-tau231 + SOST), **h** (GFAP vs GFAP + SOST) and **i** (Aβ1-42/Aβ1-40 vs Aβ1-42/Aβ1-40 + SOST).

Given the higher plasma SOST levels in the Aβ+ group compared with the Aβ− group, we investigated the predictive value of plasma SOST for brain Aβ status. We first determined the correlation between plasma SOST levels and brain SUVR using Spearman’s correlation coefficient (ρ). Intriguingly, plasma SOST levels were found to have a significant positive correlation with SUVR (ρ = 0.321, *P* = 0.001; Supplementary Fig. 2). AUC analysis was then performed to determine if SOST levels improved the diagnosis of individuals with ongoing brain deposition, either alone or in combination with other known AD-related biomarkers (GFAP, t-tau, p-tau181, p-tau231, Aβ40, Aβ42 and Aβ42/Aβ40 ratio). A base model (BM) including age, sex and *APOE* ε4 status was included in the analysis. The AUC of the base model was 0.787 (95% CI = 0.693–0.882), however, when combining the base model with the plasma SOST model, higher accuracy (AUC = 0.818, 95% CI = 0.733–0.903) was obtained compared with the base model alone, albeit not to a significant level (*P* = 0.228; Fig. 2a). The addition of SOST to the models including the BM plus known AD-related biomarkers (p-tau181, p-tau231, GFAP and Aβ42/Aβ40 ratio) also reached a higher discriminative accuracy between individuals with Aβ+ status from those with Aβ− status, while not to a significant level, neither (AUC = 0.806 vs 0.836, *P* = 0.27 for BM + p-tau181 vs BM + p-tau181 + SOST; AUC = 0.838 vs 0.852, *P* = 0.47 for BM + p-tau231 vs BM + p-tau231 + SOST; AUC = 0.916 vs 0.918, *P* = 0.69 for BM + GFAP vs BM + GFAP + SOST; AUC = 0.833 vs 0.857, *P* = 0.27 for BM + Aβ42/Aβ40 vs BM + Aβ42/Aβ40 + SOST; Fig. 2c–e). Furthermore, the addition of SOST to the models including only AD-related biomarkers (not including age, sex, APOE carriage) was also assessed and did not significantly increase the diagnostic potential for brain amyloid deposition in p-tau181 vs p-tau181 + SOST (AUC = 0.712 vs 0.759, *P* = 0.23; Fig. 2f), p-tau231 vs p-tau231 + SOST (AUC = 0.792 vs 0.816, *P* = 0.36, Fig. 2g) and GFAP vs GFAP + SOST (AUC = 0.762 vs 0.789, *P* = 0.25; Fig. 2h). Interestingly, there was a significant increase of accuracy in diagnosing brain amyloidosis upon the addition of SOST to Aβ42/Aβ40 (AUC = 0.669 vs 0.768, *P* = 0.027, Aβ42/Aβ40 vs Aβ42/Aβ40 + SOST; Fig. 2i).

## Discussion

The present study has demonstrated that the plasma level of the bone-derived protein, SOST, is positively correlated with brain Aβ load in a cognitively unimpaired older population. To our knowledge, this is the first study demonstrating the association between plasma SOST levels and preclinical AD pathological alterations related to Aβ accumulation in the brain. Importantly, the positive correlation between plasma SOST and age also highlights a possible pathophysiological relationship between osteoporosis and AD with ageing.

It is demonstrated that SOST participates in bone turnover, and for this reason has been identified as a therapeutic target for the treatment of osteoporosis^21^. In addition to its role in bone, SOST is likely to have functions in other organs beyond the skeletal system. For example, blood SOST levels correlate inversely with glomerular filtration rate after correction for age and sex in patients with chronic kidney disease^22^. In addition, higher SOST blood levels have been observed in type 2 diabetes mellitus patients compared to healthy individuals^23,24^, and there is a positive correlation between blood SOST levels and insulin resistance^25^. These findings implicate a cross-talk between bone and other organs mediated by SOST.

To further investigate the correlation of SOST plasma levels and brain Aβ load, this study also investigated the association between blood SOST and several major AD risk factors by stratifying the participants based on sex and *APOE* ε4 status. It was found that in both male and female subgroups, participants with an Aβ+ status had higher blood SOST levels compared with participants with an Aβ− status. This indicates that the association between blood SOST and brain Aβ load is independent of sex. Interestingly, we also observed high blood levels of SOST in Aβ+ individuals who did not carry the *APOE* ε4 allele, whereas there was no difference in *APOE* ε4 carriers. This observation could be attributable to the modest sample size of the *APOE* ε4 carriers, or more likely, due to the greater influence of the ApoE 4 protein compared with SOST in the development of AD. When evaluating the contribution of plasma SOST in diagnosing brain amyloidosis, it was found that in the presence of a base model including age, sex and *APOE* ε4, the addition of plasma SOST improved, albeit not to a significant level, the diagnostic accuracy of brain amyloidosis. Based on the results showing that plasma SOST levels were significantly different in Aβ− compared to Aβ+ in *APOE* ε4 non-carriers, but were similar in *APOE* ε4 carriers, we assume that the influence of the *APOE* ε4 allele overrides the influence of plasma SOST in terms of Aβ deposition.

We also evaluated the potential of combining plasma SOST with a number of AD-related biomarkers in diagnosing Aβ+ status without using the base model including age, sex and *APOE* ε4 status. Interestingly, the addition of SOST increased the validity of these biomarkers, and in particular, resulting in a significant improvement in diagnostic value for amyloid deposition when combined with the Aβ42/Aβ40 ratio alone. Further, Spearman’s correlation analysis in the whole cohort showed that plasma SOST levels were positively correlated with Aβ40 levels in the plasma. In addition, it was found that with increasing plasma SOST levels, there was an increasing trend of Aβ42 levels accompanied by a decreasing trend of the Aβ42/Aβ40 ratio in the plasma, despite such correlations not reaching statistical significance. These findings re-enforce the underlying association between plasma levels of SOST and Aβ40, Aβ42 and Aβ42/Aβ40 and amyloid deposition.

While the strengths of this study include the utilization of data from the highly characterized KARVIAH aging cohort which has been reported in previous studies^26,27^, it needs to be acknowledged that the current study has some limitations, such as the modest sample size and cross-sectional nature of the study design. Another limitation is that SOST levels in the blood may be influenced by other conditions such as chronic kidney disease and type 2 diabetes and ageing pathophysiology^11^, therefore additional inclusion/exclusion criteria taking into account other co-morbidities of the participants will need to be considered in future studies. Further studies are also required to validate these observations through longitudinal monitoring of SOST levels in an independent patient cohort. In addition, the comparison of blood SOST levels between healthy individuals and those with mild cognitive impairment or established AD would further strengthen the usefulness of SOST as an AD biomarker, and its role in AD progression. Moreover, if bone-derived SOST can have a biological effect in the brain through the Wnt/β-catenin signaling pathway, an outstanding question which has yet to be addressed relates to the extent to which it is able to cross the blood-brain barrier (BBB).

This study has demonstrated that plasma SOST levels are increased in cognitively preserved older adults at high risks of AD. A higher degree of accuracy in differentiating Aβ− and Aβ+ individuals was achieved when combining SOST blood measurements with other major AD risk factors and several putative plasma AD biomarkers, indicating that plasma SOST is a promising biomarker to assist in the detection of preclinical AD. Importantly, the association between bone-derived SOST and brain Aβ load status has drawn further attention to the importance of the ‘bone-brain axis’ and has indicated a possible common link in the pathogenesis of osteoporosis and AD.

## Methods

### Cohort

The study enrolled participants who have met a set of screening inclusion and exclusion criteria from the Kerr Anglican Retirement Village Initiative in Aging Health (KARVIAH) cohort^26,27^. The inclusion criteria comprised an age range of 65–90 years, good general health, no known significant cerebrovascular disease, fluent in English, adequate/corrected vision and hearing to enable testing, and no dementia or pathological cognitive impairment as screened by a Montreal Cognitive Assessment (MoCA) score ≥26. MoCA scores lying between 18–25 were assessed on a case-by-case basis by the study neuropsychologist following stratification of scores according to age and education. The exclusion criteria comprised the previous diagnosis of dementia based on the revised criteria from the National Institute on Aging-Alzheimer’s Association, the presence of an acute functional psychiatric disorder (including lifetime history of schizophrenia or bipolar disorder), severe or extremely severe depression (based on the Depression, Anxiety, Stress Scales; DASS), a history of stroke, and uncontrolled hypertension (systolic blood pressure [BP] >170 mm Hg or diastolic BP > 100 mm Hg).

In total, 134 volunteers met the inclusion and exclusion criteria; of these, 105 underwent neuroimaging, neuropsychometric evaluation, and blood collection; the remainder declined to undergo neuroimaging or withdrew from the study. Within these 105 participants, 100 participants were considered to have normal global cognition based on their Mini-Mental State Examination (MMSE; scores can range from 0 to 30, with higher scores indicating better cognitive function) wherein, a cut-off score <26 was employed to screen out individuals with potential early dementia. All volunteers provided written informed consent prior to participation, and the Bellberry and the Macquarie University Human Research Ethics Committees provided approval for the study.

### Brain amyloid-β load evaluation via PET

Neuroimaging was conducted within 3 months of blood collection at Macquarie Medical Imaging in Sydney. Positron emission tomography (PET) studies were conducted over a 20-min static scan (4 × 5 min frames) that was acquired 50 min after an intravenous bolus of 18F-florbetaben (FBB) administered slowly over 30 s. Brain Aβ load was calculated as the mean standard uptake value ratio (SUVR) of the frontal, superior parietal, lateral temporal, lateral occipital, and anterior and posterior cingulate regions using image processing software, CapAIBL. Participants with an SUVR ≥ 1.35 were considered to have a high brain Aβ load (Aβ+), while those with an SUVR < 1.35 were considered to have a low Aβ load (Aβ−)^26^.

### Blood collection, measurement of plasma SOST and *APOE* genotyping

All study participants fasted for a minimum of 10 h overnight prior to venesection employing standard serological methods and processing. Following blood sample processing, plasma fractions were stored at −80 °C until further testing. Plasma SOST concentration was measured using the human SOST ELISA kit (BI-20492, Biomedica, Wien, Austria) according to the manufacturer’s instructions. In brief, Assay buffer was added into pre-coated plates followed with addition of plasma samples in duplicate. Samples were then incubated in wells simultaneously with biotinylated antibody overnight (18–24 h) at room temperature (18–24 °C) and washed 5x prior to incubation with conjugation buffer for 1 h at room temperature in the dark. The conjugation buffer was aspirated and wells washed 5× prior to a 30-min incubation with substrate buffer in the dark. At the end of the incubation, stop solution was added into each well and the absorbance measured immediately at 450 nm. The average signal coefficient of variation (%) of all data is 5.57%. A standard curve was constructed from the absorbance values of the standards. Plasma SOST concentrations of the samples were obtained from the standard curve. Apolipoprotein E (*APOE*) genotype was determined from purified genomic DNA extracted from 0.5 mL whole blood. Each sample was genotyped for the presence of the three *APOE* variants (ε2, ε3 and ε4) based on TaqMan SNP genotyping assays for rs7412 (C 904973) and rs429358 (C3084793) as per the manufacturer’s instructions (AB Applied Biosystems by Life Technologies, Scoresby, VIC, Australia). Five percent of the samples were genotyped in duplicate and 100% inter- and intra-assay concordance was observed.

### Blood biomarkers analysis

The single molecule array (Simoa) platform was used to measure protein biomarker concentrations in EDTA plasma specimens. GFAP, t-tau and NFL concentrations were measured using the Neurology 4-Plex A kit (QTX-102153, Quanterix, MA, USA), while p-tau181 and p-tau231 concentrations were measured with in-house assays developed at the University of Gothenburg, Sweden^15,17^. Calibrators and samples were run in duplicates in all assays. Two internal controls provided with the Simoa kits were included in duplicate at the beginning of each experiment. Plasma Aβ concentrations were measured using the Amyblood test that was developed at Amsterdam University Medical Center in collaboration with ADx NeuroSciences (Ghent, Belgium), on the Simoa platform (HDx instrument, Quanterix)^27,28^.

### Statistical analyses

Student’s *t* tests or Chi-square tests were employed to compare descriptive statistics including means and standard deviations calculated for Aβ− and Aβ+ groups. Continuous variables between Aβ− and Aβ+ groups were compared by employing linear models after correcting for covariates age, sex, and *APOE* ε4 carrier status. To better approximate normality and variance homogeneity, dependent variables were transformed to natural log as required. Correlations between continuous variables were investigated using the Spearman correlation coefficient (ρ). Predictive models and receiver-operating characteristic (ROC) curves constructed from the logistic scores were evaluated by using logistic regression with Aβ−/+ as response. All data analyses and visualization were carried out using IBM^®^ SPSS (v27) or Stata MP (v17.0) and GraphPad Prism (v8), respectively.

### Reporting summary

Further information on research design is available in the Nature Research Reporting Summary linked to this article.

## Supplementary information

Supplementary figures

Reporting summary

## Data Availability

All data generated or analyzed during this study are included in this paper or the supplementary materials. Additional data are available from the authors upon reasonable request.
