# Supplementary material for: Elevated plasma sclerostin is associated with high brain amyloid-β load in cognitively normal older adults
Source: NPJ Aging. 2023 Sep 4;9(1):17. doi: 10.1038/s41514-023-00114-4 (PMC10477312; doi:10.1038/s41514-023-00114-4)

Supplementary Content

Supplementary Figures:

Supplementary Figure 1. Spearman's analysis for the correlation of plasma sclerostin with NFL, GFAP, t-tau, p-tau181 and p-tau231, Aβ42, Aβ40 and Aβ42/Aβ40 in all participants.

Supplementary Figure 2. Spearman's analysis for the correlation of plasma sclerostin and SUVR.

Supplementary Figure 1. Spearman's analysis for the correlation of plasma sclerostin with NFL, GFAP, t-tau, p-tau181 and p-tau231, Aβ42, Aβ40 and Aβ42/Aβ40 in all participants. Schematic representation of unadjusted correlation coefficients (ρ) and *P* values between sclerostin and AD-related biomarkers were calculated using Spearman's correlation.

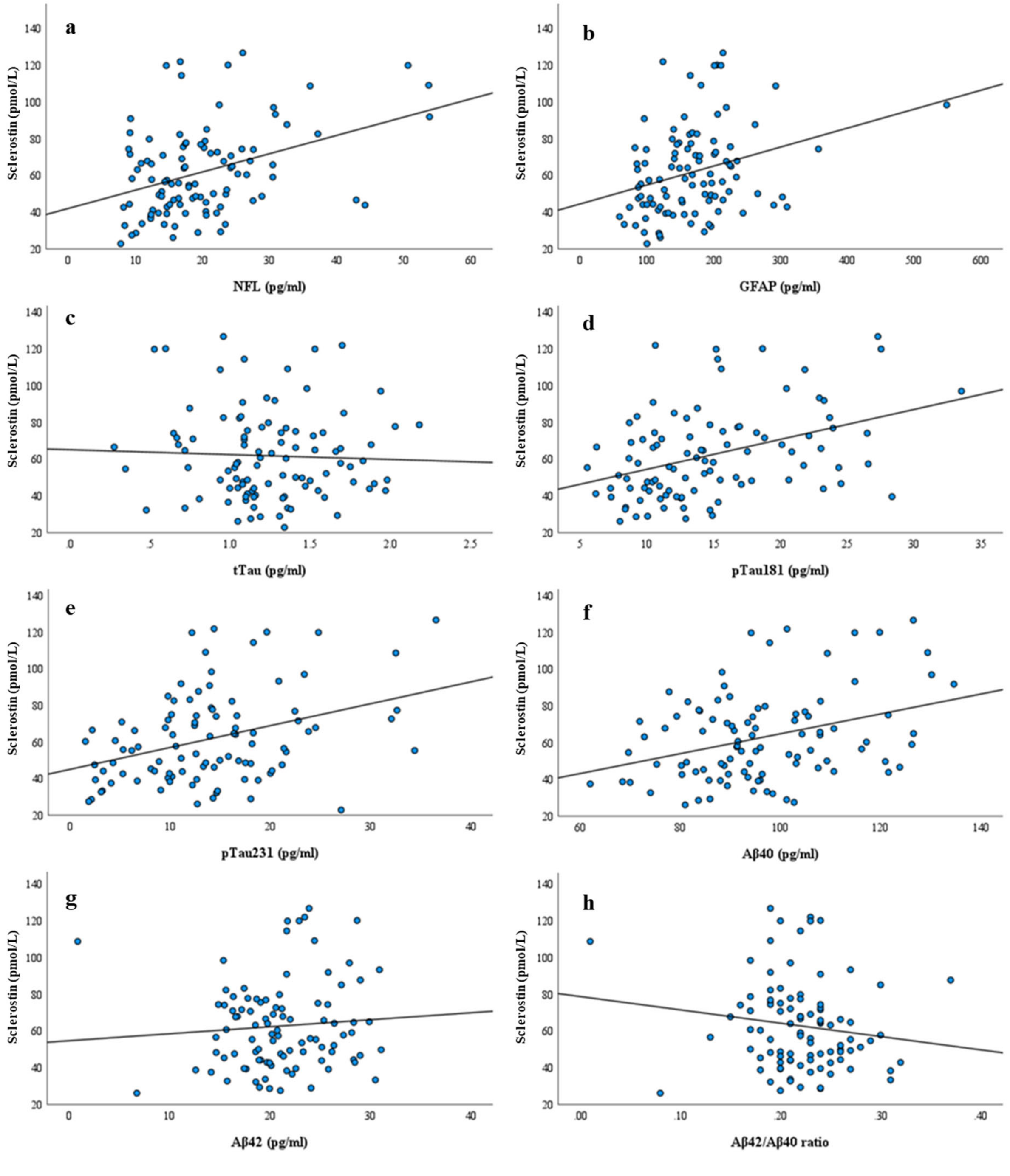

Supplementary Figure 2. Association between plasma sclerostin and SUVR. Correlation coefficients (ρ) and *P* values were calculated using Spearman's correlation analysis. SUVR, standard uptake value ratio.

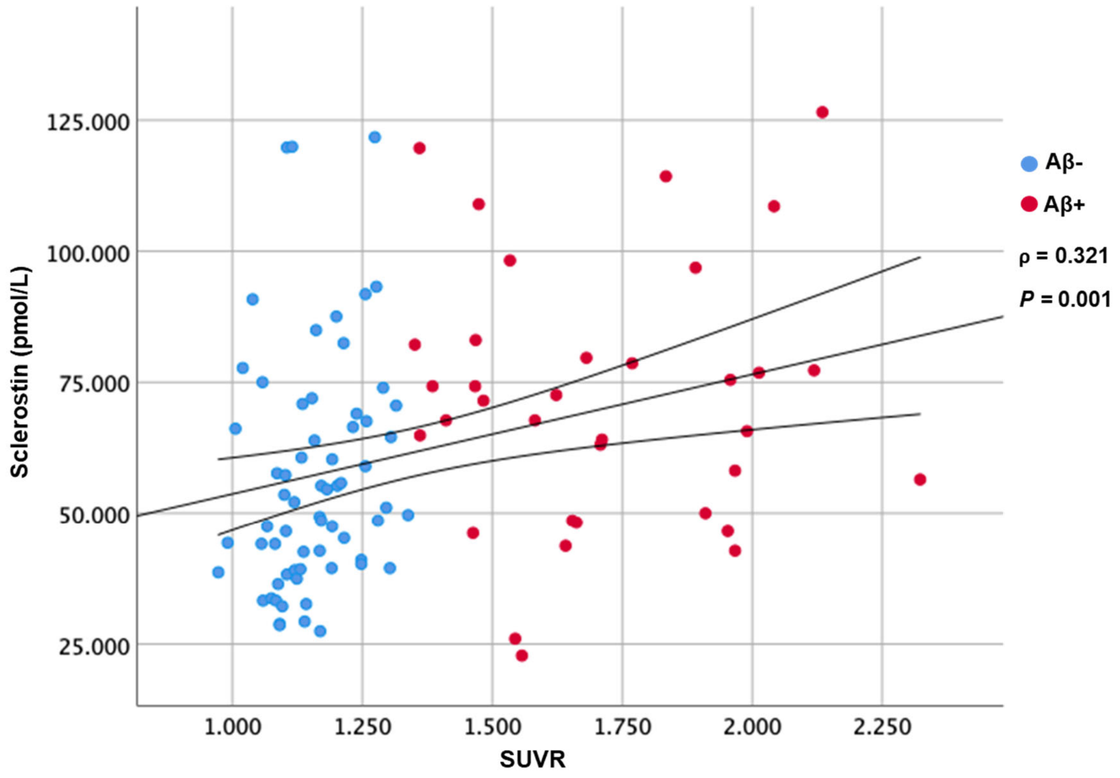

Supplement: Supplementary file 1 — Supplementary figures [file 41514_2023_114_MOESM1_ESM.pdf]
