# Supplementary material for: Elevated plasma sclerostin is associated with high brain amyloid-β load in cognitively normal older adults
Source: NPJ Aging. 2023 Sep 4;9(1):17. doi: 10.1038/s41514-023-00114-4 (PMC10477312; doi:10.1038/s41514-023-00114-4)
Supplement: Supplementary file 2 — Reporting summary [file 41514_2023_114_MOESM2_ESM.pdf]

nature portfolio

Corresponding author(s): Minghao Zheng

Last updated by author(s): May 29, 2023

Reporting Summary

Nature Portfolio wishes to improve the reproducibility of the work that we publish. This form provides structure for consistency and transparency in reporting. For further information on Nature Portfolio policies, see our Editorial Policies and the Editorial Policy Checklist.

Statistics

For all statistical analyses, confirm that the following items are present in the figure legend, table legend, main text, or Methods section.

n/a	Confirmed	
☐	☒	The exact sample size (*n*) for each experimental group/condition, given as a discrete number and unit of measurement
☐	☒	A statement on whether measurements were taken from distinct samples or whether the same sample was measured repeatedly
☐	☒	The statistical test(s) used AND whether they are one- or two-sided<br>*Only common tests should be described solely by name; describe more complex techniques in the Methods section.*
☐	☒	A description of all covariates tested
☐	☒	A description of any assumptions or corrections, such as tests of normality and adjustment for multiple comparisons
☐	☒	A full description of the statistical parameters including central tendency (e.g. means) or other basic estimates (e.g. regression coefficient) AND variation (e.g. standard deviation) or associated estimates of uncertainty (e.g. confidence intervals)
☐	☒	For null hypothesis testing, the test statistic (e.g. *F*, *t*, *r*) with confidence intervals, effect sizes, degrees of freedom and *P* value noted<br>*Give P values as exact values whenever suitable.*
☒	☐	For Bayesian analysis, information on the choice of priors and Markov chain Monte Carlo settings
☒	☐	For hierarchical and complex designs, identification of the appropriate level for tests and full reporting of outcomes
☐	☒	Estimates of effect sizes (e.g. Cohen's *d*, Pearson's *r*), indicating how they were calculated

Our web collection on statistics for biologists contains articles on many of the points above.

Software and code

Policy information about availability of computer code

Data collection	Microsoft Office 365 (Excel, Semi-Annual Enterprise Channel), image processing software, CapAIBL;
Data analysis	IBM® SPSS (v27), Stata MP (v17.0) and GraphPad Prism (v8)

For manuscripts utilizing custom algorithms or software that are central to the research but not yet described in published literature, software must be made available to editors and reviewers. We strongly encourage code deposition in a community repository (e.g. GitHub). See the Nature Portfolio guidelines for submitting code & software for further information.

Data

Policy information about availability of data

All manuscripts must include a data availability statement. This statement should provide the following information, where applicable:

- Accession codes, unique identifiers, or web links for publicly available datasets
- A description of any restrictions on data availability
- For clinical datasets or third party data, please ensure that the statement adheres to our policy

All data generated or analyzed during this study are included in this paper or the supplementary materials. Additional data are available from the authors upon reasonable request.

Research involving human participants, their data, or biological material

Policy information about studies with human participants or human data. See also policy information about sex, gender (identity/presentation), and sexual orientation and race, ethnicity and racism.

Reporting on sex and gender	Totally 100 participants were enrolled in this study, among these participants, 32 were male and 68 were female.
Reporting on race, ethnicity, or other socially relevant groupings	None.
Population characteristics	Participants with an standard uptake value ratio (SUVR) ≥ 1.35 were considered to have a high brain Aβ load (Aβ+), while those with an SUVR < 1.35 were considered to have a low Aβ load (Aβ-). No significant difference was observed in terms of age, sex, MMSE and BMI between the Aβ- and Aβ+ groups. There was a higher ratio of APOE ε4 carriage in the Aβ+ group (45.7%) compared with Aβ- group (7.7%; P < 0.001). By design, the SUVR values in the Aβ+ group (1.71 ± 0.26) were significantly higher than the Aβ- group (1.16 ± 0.09; P < 0.001).
Recruitment	The study enrolled participants who have met a set of screening inclusion and exclusion criteria from the Kerr Anglican Retirement Village Initiative in Aging Health (KARVIAH) cohort. The inclusion criteria comprised an age range of 65-90 years, good general health, no known significant cerebrovascular disease, fluent in English, adequate/corrected vision and hearing to enable testing, and no dementia or pathological cognitive impairment as screened by a Montreal Cognitive Assessment (MoCA) score ≥ 26. MoCA scores lying between 18-25 were assessed on a case-by-case basis by the study neuropsychologist following stratification of scores according to age and education. The exclusion criteria comprised the previous diagnosis of dementia based on the revised criteria from the National Institute on Aging-Alzheimer's Association, the presence of an acute functional psychiatric disorder (including lifetime history of schizophrenia or bipolar disorder), severe or extremely severe depression (based on the Depression, Anxiety, Stress Scales; DASS), a history of stroke, and uncontrolled hypertension (systolic blood pressure [BP] >170 mm Hg or diastolic BP >100 mm Hg).<br>In total, 134 volunteers met the inclusion and exclusion criteria; of these, 105 underwent neuroimaging, neuropsychometric evaluation, and blood collection; the remainder declined to undergo neuroimaging or withdrew from the study. Within these 105 participants, 100 participants were considered to have normal global cognition based on their Mini-Mental State Examination (MMSE; scores can range from 0 to 30, with higher scores indicating better cognitive function) wherein, a cut-off score < 26 was employed to screen out individuals with potential early dementia.
Ethics oversight	All volunteers provided written informed consent prior to participation, and the Bellberry and the Macquarie University Human Research Ethics Committees provided approval for the study.

Note that full information on the approval of the study protocol must also be provided in the manuscript.

Field-specific reporting

Please select the one below that is the best fit for your research. If you are not sure, read the appropriate sections before making your selection.

☒ Life sciences ☐ Behavioural & social sciences ☐ Ecological, evolutionary & environmental sciences

For a reference copy of the document with all sections, see nature.com/documents/nr-reporting-summary-flat.pdf

Life sciences study design

All studies must disclose on these points even when the disclosure is negative.

Sample size	Brain imaging and blood samples were obtained from 100 participants, with demographic and clinical characteristics
Data exclusions	No data were excluded from the analyses.
Replication	Calibrators and samples were run in duplicates in all assays.
Randomization	Participants with an standard uptake value ratio (SUVR) ≥ 1.35 were considered to have a high brain Aβ load (Aβ+), while those with an SUVR < 1.35 were considered to have a low Aβ load (Aβ-).
Blinding	Investigators detecting blood biomarkers were blinded to group allocation.

Reporting for specific materials, systems and methods

We require information from authors about some types of materials, experimental systems and methods used in many studies. Here, indicate whether each material, system or method listed is relevant to your study. If you are not sure if a list item applies to your research, read the appropriate section before selecting a response.

Materials & experimental systems

n/a	Involved in the study
☒	☐ Antibodies
☒	☐ Eukaryotic cell lines
☒	☐ Palaeontology and archaeology
☒	☐ Animals and other organisms
☒	☐ Clinical data
☒	☐ Dual use research of concern
☒	☐ Plants

Methods

n/a	Involved in the study
☒	☐ ChIP-seq
☒	☐ Flow cytometry
☒	☐ MRI-based neuroimaging
